# Functional Activity of TDP-43: A Direct Biomarker for ALS

**DOI:** 10.3390/bios16080446

**Published:** 2026-08-17

**Authors:** Kirti Shila Sonkar, Vito Levi D’Ancona, Jade Cramp, Hannah Shilling, Ellie Giles, Tyler Howell-Bray, Becky Fillingham, Merit E. Cudkowicz, Avindra Nath, Jeffrey D. Rothstein, Robert Bowser, Barbara Borroni, James D. Berry, Ghazaleh Sadri-Vakili, Emanuele Buratti, Ian P. Thrippleton

**Affiliations:** 1 Nemdx, Auburndale, MA 02466, USA; vito@nemdx.com (V.L.D.); jade@qbiotix.com (J.C.); hannah@qbiotix.com (H.S.); ellie@qbiotix.com (E.G.); t.howell-bray@hull.ac.uk (T.H.-B.); 2 Qbiotix Developments Ltd., Discovery Park, Sandwich, Kent CT13 9FE, UK; 3 School of Environmental and Life Science, Faculty of Science and Engineering, University of Hull, Hull HU6 7RX, UK; 4 Healey & AMG Centre for ALS, Mass General Brigham, Boston, MA 02114, USA; bfillingham@mgh.harvard.edu (B.F.); cudkowicz.merit@mgh.harvard.edu (M.E.C.); jdberry@mgh.harvard.edu (J.D.B.); gsadrivakili@mgh.harvard.edu (G.S.-V.); 5 National Institute of Neurological Disorders and Stroke (NINDS), NIH, Bethesda, MD 20892, USA; avindra.nath@nih.gov; 6 School of Medicine, Johns Hopkins University, Baltimore, MD 21287, USA; jrothst1@jh.edu; 7 Barrow Neurological Institute, Dignity Health, Phoenix, AZ 85013, USA; robert.bowser@commonspirit.org; 8 Department of Clinical and Experimental Sciences, University of Brescia, 25123 Brescia, Italy; barbara.borroni@unibs.it; 9 IRCCS Centro Istituto Fatebenefratelli San Giovanni di Dio, 25125 Brescia, Italy; 10 Molecular Pathology Group, International Centre for Genetic Engineering and Biotechnology (ICGEB), 34149 Trieste, Italy; emanuele.buratti@icgeb.org

**Keywords:** TDP-43, amyotrophic lateral sclerosis, serum biomarker, RNA-binding function, hTR-FRET, functional proteomics

## Abstract

TDP-43 dysfunction is a defining feature of amyotrophic lateral sclerosis (ALS), yet no biofluid biomarker directly measures its functional activity. We developed a serum-based homogeneous time-resolved FRET (hTR-FRET) assay that quantifies TDP-43 RNA binding activity using synthetic UU-rich RNA probes. We analyzed 1080 serum samples from controls, sporadic ALS, and genetic subgroups (C9orf72, SOD1) across multiple biorepositories. Cross-sectionally, TDP-43 functional activity was elevated in ALS (mean 390 a.u.) versus controls (302 a.u.), yielding AUC = 0.79. Genotype means were 392 a.u. (sporadic), 382 a.u. (C9orf72), and 323 a.u. (SOD1); a 366 a.u. threshold achieved 95% specificity against controls. Longitudinally, Target ALS showed a modest but significant inverse correlation between TDP-43 activity and ALSFRS-R, while other cohorts exhibited similar non-significant trends. Elevated signal in serum likely reflects increased extracellular release of probe-competent TDP-43 species during cell death and exosomal shedding, rather than restored intracellular nuclear splicing function. This assay provides a proof-of-concept platform for the direct functional measurement of probe-competent TDP-43 species in serum. While it demonstrates moderate group-level discrimination, individual diagnostic performance requires prospective validation. The assay may support exploratory applications in genotype stratification and progression monitoring in future clinical studies.

## 1. Introduction

TAR DNA-binding protein 43 (TDP-43) is a ubiquitously expressed, multifunctional DNA/RNA binding protein critical for essential cellular processes, including transcriptional regulation, pre-mRNA splicing, mRNA stability, and microRNA biogenesis [1,2,3,4,5]. Its function is critically dependent on its distinct domain structure, featuring two RNA binding domains (RBD) that mediate high-affinity binding to UU-rich RNA sequences, and a C-terminal domain that is intrinsically disordered and prone to aggregation [2,6]. Under physiological conditions, TDP-43 is predominantly nuclear but shuttles between the nucleus and cytoplasm to perform its duties in RNA metabolism [7,8]. The central role of TDP-43 in neurodegeneration was unveiled when it was identified as the primary constituent of the pathological cytoplasmic inclusions in the majority of Amyotrophic Lateral Sclerosis (ALS) cases and a significant subset of Frontotemporal Dementia (FTD) cases [9] and recently reviewed by [3,10]. This pathology, termed TDP-43 proteinopathy, is characterized by the nuclear depletion of TDP-43, its cytoplasmic mislocalization, hyper-phosphorylation, and cleavage into insoluble aggregates [7]. These aggregates are not merely a hallmark of disease but are directly implicated in pathogenic cascades through a loss of normal nuclear function and a potential gain of toxic function in the cytoplasm [1]. The critical loss of TDP-43’s splicing repression function, for instance, has been shown to lead to aberrant cryptic exon inclusion in transcripts like UNC13A and STMN2, genes vital for neuronal survival [7,11]. TDP-43 pathology is now recognized as a common feature across a spectrum of disorders, like ALS, FTD, Alzheimer’s Disease and limbic-predominant age-related TDP-43 encephalopathy [4,8,10]. Despite its clear pathological significance, a significant gap persists in our ability to measure the functional state of TDP-43 in vivo during a patient’s life [12]. While emerging biomarkers, such as cryptic peptides (e.g., HDGFL2), are beginning to reflect TDP-43 dysfunction, they are indirect readouts and not a direct measure of the protein’s core RNA-binding function.

While conventional immunoassays and modern high-throughput proteomic platforms excel at quantifying static protein abundance or specific post-translational modifications [1,8] they are inherently blind to biochemical competence. In the case of TDP-43, pathogenicity is driven not merely by its presence, but by its loss of nuclear RNA-binding function [1] and cytoplasmic mislocalization. Therefore, a biomarker that directly captures probe-competent TDP-43 activity addresses a critical gap in measuring the protein’s functional state in vivo. Moreover, the use of different biofluid matrices, particularly plasma versus serum, has led to inconsistent results, with evidence suggesting that plasma anticoagulants and fibrinogen complexes can interfere with TDP-43 measurements [13]. A biomarker that detects extracellular, probe-competent TDP-43 species in serum would therefore address a critical gap. The assay described here measures TDP-43’s ability to recognize and bind synthetic UU-rich RNA motifs in a biofluid context. It reflects circulating probe-competent protein burden resulting from cellular degeneration and exosomal release and does not report restored nuclear RNA-binding function or splicing competence. Systemic availability of TDP-43 in bloodstream biofluids occurs via active exosomal secretion, extracellular vesicle (EV) shedding, progressive blood–brain barrier (BBB) hyperpermeability during neurodegeneration, and peripheral tissue turnover [7].

**Figure 1:**
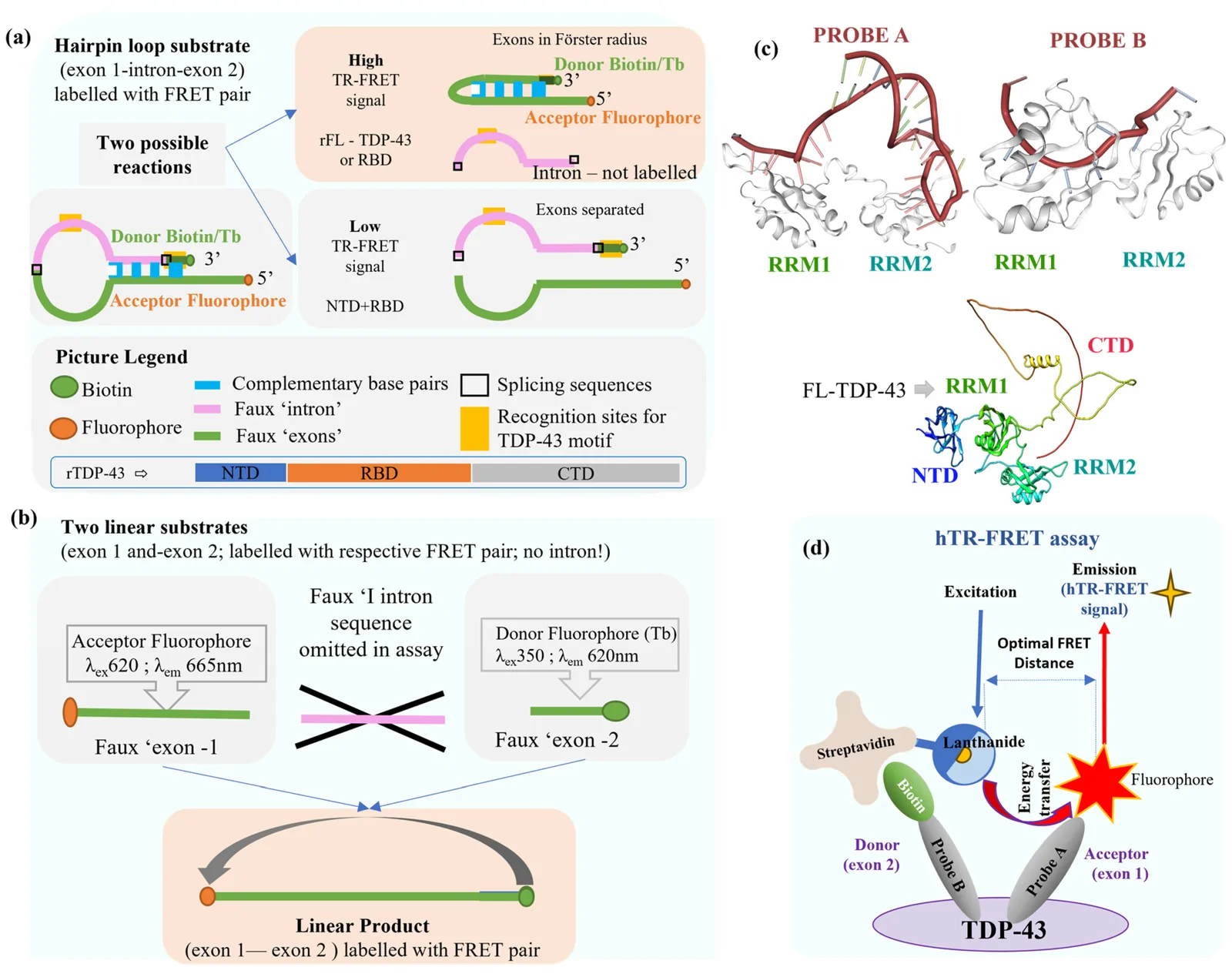
Design, structural basis, and physical mechanics of the TDP-43 functional hTR-FRET assay: (**a**) Hairpin-loop substrate architecture containing faux exons 1 and 2 linked by a faux intron, displaying differential FRET states based on TDP-43 engagement. (**b**) Optimized linear dual-probe bridging assay omitting the intron loop to eliminate nuclease susceptibility and isolate RNA-bridging activity. (**c**) Displaying dual-probe (Probe A and Probe B) engagement across tandem RNA recognition motifs (RRM1/2), Structural model of full-length TDP-43 (NTD, RRM1, RRM2, CTD) (**d**) Biophysical mechanism of time-resolved gated energy transfer upon TDP-43-mediated probe alignment within the Förster radius ($R_{0}\approx 7\;\mathrm{nm}$).

Foundational studies defined the UU-rich sequence grammar that underlies TDP-43’s RNA specificity, providing a molecular blueprint for a function-based assay [14]. Building on this knowledge, we constructed a nucleotide sequence fulfilling the TDP-43 motif requirements. We established a homogeneous hTR-FRET assay in which the TDP-43 motif dependent probe generates a measurable signal that reflects its binding activity in context (Figure 1a). By introducing a direct functional biomarker for TDP-43, the work described here aims to bridge the gap between molecular pathology and clinical application, supporting applications in population-level discrimination of ALS from controls and exploratory longitudinal trends. The ALS Functional Rating Scale Revised (ALSFRS-R) is a validated clinical tool used to quantify motor function decline and disease progression, which we utilized to correlate biochemical changes with clinical severity.

## 2. Materials and Methods

### 2.1. Study Design

Analytical decisions were pre-specified prior to unblinding: single-hypothesis testing (TDP-43 RNA-bridging competence as the outcome), run triplicate protocol with CV >15% exclusion, and pre-defined LoB/LoD thresholds (see Section 2.6). All experiments prospectively tested a single hypothesis: that a synthetic UU-rich RNA substrate could convert the functional state of TDP-43 in human serum into a quantifiable optical signal.

### 2.2. Serum Samples

The study integrates data from multiple sources, as summarized in Table 1. The dataset includes ALS samples from NEALS (AIM, Bio1, Bio2, Bio3, ALS biorepository), NIH/NINDS repositories, the Spedali Civili Hospital of Brescia clinical biobank, Answer ALS, Target ALS, and AD and FTD samples from iSpecimen, totaling 1080 serum samples. Each sample was processed for the hTR-FRET analysis under uniform analytical conditions, and every plate incorporated reference controls to ensure cross-site comparability. The assay design is mechanism-driven, leveraging known TDP-43 RNA-binding domain geometry and FRET physics to convert functional RNA engagement into a quantifiable optical signal.

### 2.3. IRB and Sample Collection Information

Samples included in this analysis were drawn from prospectively designed biorepositories and longitudinal studies but analyzed retrospectively for this cross-sectional/exploratory study, spanning mixed populations and multiple cohorts across different geographic regions. All sample collections were carried out under protocols approved by the Mass General Brigham Institutional Review Board (IRB), Barrow Neurological Institute IRB, NIH/NINDS and Spedali Civili Hospital of Brescia, Italy, relevant local IRBs at each relevant participating study site, and TARGET ALS (samples from 2 patients were considered compromised and not included in our analysis). Prior to undergoing any data collection or study procedures, all participants underwent an informed consent process and provided written informed consent.

#### 2.3.1. Human Plasma and Serum Samples

A random sampling of de-identified plasma and serum from sALS (102 sALS) and healthy controls (10 HC) were obtained from the Northeast Amyotrophic Lateral Sclerosis Consortium (NEALS) Biofluid Repository at Mass General Brigham and the Barrow Neurological Institute, a shared repository containing more than 100,000 biofluid samples from people with ALS and healthy controls for use by the ALS research community. The samples and associated clinical data were collected between 2008 and 2023 from participants in multi-site research studies. Of the 102 sALS, 94 had plasma and serum samples available from two to three visits; one had serum only and two had plasma only available. Serum was also provided from a single visit for healthy controls. Across all studies, serum samples were collected using standard red-top tubes (no SST gel additives), allowed to clot fully at room temperature for 30 min, and then centrifuged (1750× *g*, 10 min). The supernatant was carefully aliquoted without disturbing the platelet/cell pellet. The 10 plasma samples were collected using EDTA tubes and blood for serum using standard red-top tubes and centrifuged immediately. Vacutainers were centrifuged at 1750× *g* for 10 min, and supernatants aliquoted into cryovials and frozen at −70 °C to −80 °C within two hours of collection. To preserve native structural conformations and avoid pre-analytical bias associated with freeze–thaw-induced protein precipitation [15], all samples were stored below −70 °C, with zero freeze–thaw cycles. All test aliquots were thawed once immediately prior to hTR-FRET processing. Formal multi-cycle freeze–thaw and matrix interference testing (hemolysis, lipemia) were not conducted on this retrospective cohort and are designated for prospective analytical validation. Residual platelet-derived TDP-43 contamination cannot be excluded and is acknowledged as a limitation. Future studies will evaluate SST vs. red-top tube impacts on assay signal.

#### 2.3.2. Serum vs. Plasma

Matrix Selection: Serum was selected over plasma based on mechanistic considerations. Plasma contains polymeric fibrinogen and anticoagulants (EDTA, heparin, citrate) additives that bind TDP-43, attenuate free protein pools [13,16], and introduce nonspecific FRET quenching and ionic variability that disrupt donor–acceptor coupling. Serum, by contrast, better represents the physiologically relevant soluble fraction of TDP-43 released from cells and exosomes. Consistent with NEALS and Answer ALS protocols, all samples in this study were serum-derived to avoid matrix-related artifacts and maintain compatibility with reference datasets. Empirical testing of matched serum–plasma pairs from ten donors confirmed this choice: serum produced stable biphasic kinetic traces, whereas plasma yielded flat or erratic profiles. Taken together, serum was adopted as the exclusive matrix.

#### 2.3.3. Specificity Validation

Specificity was validated through orthogonal experimental axes: (1) chemistry dependence (RNA but not DNA substrates produced the biphasic kinetic trace), and (2) protein identity (full-length (1-414; TDP-43 FL) and NTD + RBD construct showed dip and rise activity, whereas RBD-only construct (without N terminus) showed only the rise phase. With this pattern, we can conclude that the TDP-43 N-terminus domain is responsible for the initial dip phase. This validation established that the fluorescence increase cannot arise from random molecular crowding or nonspecific nucleoprotein complexes.

### 2.4. RNA Substrate Design

#### 2.4.1. Rationale

TDP-43 binds RNA through its tandem RNA binding domain (RBD) that sandwiches UU-rich sequences into a shallow hydrophobic cleft [17]. These insights were used to design a pair of synthetic RNA exons, where one carries a UU motif, flanked by stabilizing spacers that mimic natural exon junctions. Both oligonucleotides were 2′-O-methyl modified to improve nuclease resistance without altering hydrogen bonding geometry.

#### 2.4.2. Architecture and Labeling

To accommodate distinct diagnostic readouts, two complementary biosensor architectures were synthesized with 2′-O-methyl backbone modifications:

1. Hairpin Loop Substrate: To ensure post-cleavage strand separation and FRET loss, hairpin probes were engineered with specific thermodynamic parameters. The intact probe forms a stable hairpin stem at 37 °C ($T_{m}\approx 58\;{}^{\circ}C$), whereas post-cleavage fragments exhibit a $T_{m}<24\;{}^{\circ}C$, rendering post-cleavage hybridization thermodynamically unfavorable and driving rapid dissociation. All synthesized probes feature 2′-O-methyl backbone modifications. The hairpin loop substrate sequences are:
  **Splicing Substrate: **5’-taaaaaaaacgaacactcccaaaaaaaaacaaaggcttcATCTTTTTTTTTTTTACTTTTTTTTTTTTCTTTGTtttttttta-3’;
  **Spliced Fragment: **5’-ATCTTTTTTTTTTTTACTTTTTTTTTTTTTTTGT-3’;
  **Spliced Product: **5’-taaaaaaaacgaacactcccaaaaaaaaacaaagcttcgtttttttta-3’.
2. Two Linear Substrates: To quantify circulating TDP-43 protein abundance, a two-component linear bridging system was developed:

Acceptor Probe (Probe A/Faux Exon 1): 5’-[Atto 620]-UAAAAAAAACGAACACUCCCAAAAAAAAACAAAGGUUUCG- 3’ labeled at the 5’ terminus with Atto 620 fluorophore. Acceptor emission is detected at 665±10 nm to eliminate spectral overlap with donor emission peaks.

Donor Probe (Probe B/Faux Exon 2): 5’—UUUUUUUUUUA—[Biotin]—3’ labeled at the 3’ terminus with biotin, which couples to a streptavidin–terbium cryptate donor.

When TDP-43 bridges the UU motifs, the probes move within the Förster radius (R_0 ≈ 7 nm), enabling distance-dependent energy transfer [18]. Using the Förster relationship $E=\frac{1}{1+{\left(r/R_{0}\right)}^{6}}$, this setup provides a homogeneous, physics-driven assay suitable for detecting analytes in impure biological samples. Complete excitation and emission spectra for the donor and acceptor probes are provided in Figure S1.

#### 2.4.3. Control Substrates

In addition to the UU-based probe, three categories of controls were employed: DNA versions of the probes (lacking 2′-OH) to test chemistry dependence, and scrambled competitors (“cold” oligos) to test sequence dependence and truncated proteins.

### 2.5. hTR-FRET Detection Physics

Time-resolved Förster resonance energy transfer (hTR-FRET) was selected for its ability to separate long-lived donor emission from short-lived biofluid autofluorescence, enabling high-sensitivity serum assays in the pg/mL–fg/mL range.

#### 2.5.1. Physical Basis

FRET transfers energy from an excited donor to an acceptor via dipole–dipole interaction, with efficiency highly dependent on proximity [18]. To achieve sensitive detection in complex fluids, we employed lanthanide-based probes in a homogeneous format. A streptavidin–terbium cryptate donor was chosen for its millisecond-scale lifetime (≈1.5–1.6 ms), compared to the nanosecond lifetimes of endogenous fluorophores. By delaying detection (Δt = 60 µs) and integrating over a 400 µs gate, background fluorescence was effectively eliminated. The signal was quantified as the ratio of acceptor to donor emission (665 nm/620 nm), normalized to the per-plate healthy pooled serum (HPS) control mean, and reported in arbitrary units (a.u.). Positive control: 50 nM recombinant FL-TDP-43 (expected 420–480 a.u.); negative control: probe blank without protein (expected <200 a.u.). These benchmarks were included on every plate to define assay dynamic range and normalization reference. Fluorescence lifetime acquisition is not available on the BMG VANTAstar platform used. While lifetime-based FRET analysis would offer higher precision, intensity-based ratiometric detection was employed due to platform constraints. Error propagation for the FRET ratio follows standard ratiometric analysis:

$$\frac{\Delta R}{R}=\sqrt{{\left(\frac{\Delta I_{665}}{I_{665}}\right)}^{2}+{\left(\frac{\Delta I_{620}}{I_{620}}\right)}^{2}}$$

which forms the basis of the reported intra-assay CV.

#### 2.5.2. Instrumentation

All assays were performed on BMG Labtech ClARIOstar Plus and VANTAstar F readers in TR/F mode with the following parameters: excitation at 350 ± 10 nm, emission 1 at 620 ± 10 nm, and emission 2 at 665 ± 10 nm with a delay of 60 µs, integration at 400 µs, 20 flashes per read, 25 °C, and top optics. The plates were Corning 384-well low-volume white microplates. Each sample was run in triplicate, with column-wise inclusion of healthy pooled serum (HPS) and blank controls.

### 2.6. Assay Workflow and Quality Control

Each sample run consisted of 5 µL triplicate wells per sample (15 µL total) and two incubation phases at 25 °C for 1 h each to allow equilibrium binding and conformational closure. Triplicates with a coefficient of variation (CV) > 15% were automatically excluded. Analytical thresholds were calculated as: limit of blank (LoB) = mean blank + 1.645 × SD blank; limit of detection (LoD) = LoB + 1.645 × SD low. Across 28 plates, intra-assay CV averaged 4.4% and inter-plate CV 15%, meeting or exceeding clinical laboratory precision standards (Figure S2). Plates failing to meet the control criteria were removed from analysis.

### 2.7. Statistical Analyses

Datasets were processed in R 4.3 using packages tidyverse, rstatix, and FactoMineR. Primary outcome: mean hTR-FRET values (a.u.) per sample. Group comparisons employed Welch’s *t*-tests; effect sizes are expressed as Cohen’s d. Precision: CV = 100 × SD/mean. Diagnostic performance (sensitivity, specificity, PPV, and NPV) across optimal cutoffs determined by Youden’s Index was evaluated using Receiver Operating Characteristic (ROC) analysis, with 95% confidence intervals (95% CIs). To control for clinical and technical confounders, multivariable logistic regression and linear mixed-effects models adjusted for fixed covariates (age, sex, disease duration, baseline ALSFRS-R, cohort source, storage duration) and plate-batch random intercepts. Two-tailed significance was set at p<0.05.

### 2.8. Longitudinal Cohort Designs

To assess temporal stability and progression, we analyzed serial serum samples from NEALS, Target ALS, and Answer ALS repositories. Participants (ALS) had 2–6 visits over 6–24 months, linked by unique identifiers. All samples were processed using the same hTR-FRET protocol and calibration curves. Survival data were derived from biorepository clinical databases and linked via unique identifiers. Incomplete follow-up is acknowledged as a limitation. Changes in TDP-43 activity were expressed as ΔFRET = FRET (visit *n*)—FRET (baseline, visit 1). Sensitivity analyses using visit-to-visit changes (FRET (visit *n*)—FRET (visit *n* − 1)) yielded similar conclusions. Statistical analysis used paired *t*-tests for within-subject comparisons and mixed-effects linear models to estimate progression slopes by diagnostic group. All assay runs were performed blinded to diagnostic status. Cases and controls were randomly distributed across plates to minimize batch effects.

## 3. Results

### 3.1. A Novel hTR-FRET Assay Measures the Functional Activity of TDP-43

To quantify the functional, probe-competent state of circulating TDP-43 in serum biofluids, we engineered a homogeneous time-resolved Förster resonance energy transfer (hTR-FRET) assay. The core analytical readout measures the dynamic amplitude of probe-bridging activity, quantified as the ratiometric FRET signal relative to baseline reference controls.

During initial assay design, we utilized a single-molecule hairpin-loop substrate designed to satisfy TDP-43 structural binding requirements (Figure 1a).

**Figure 2:**
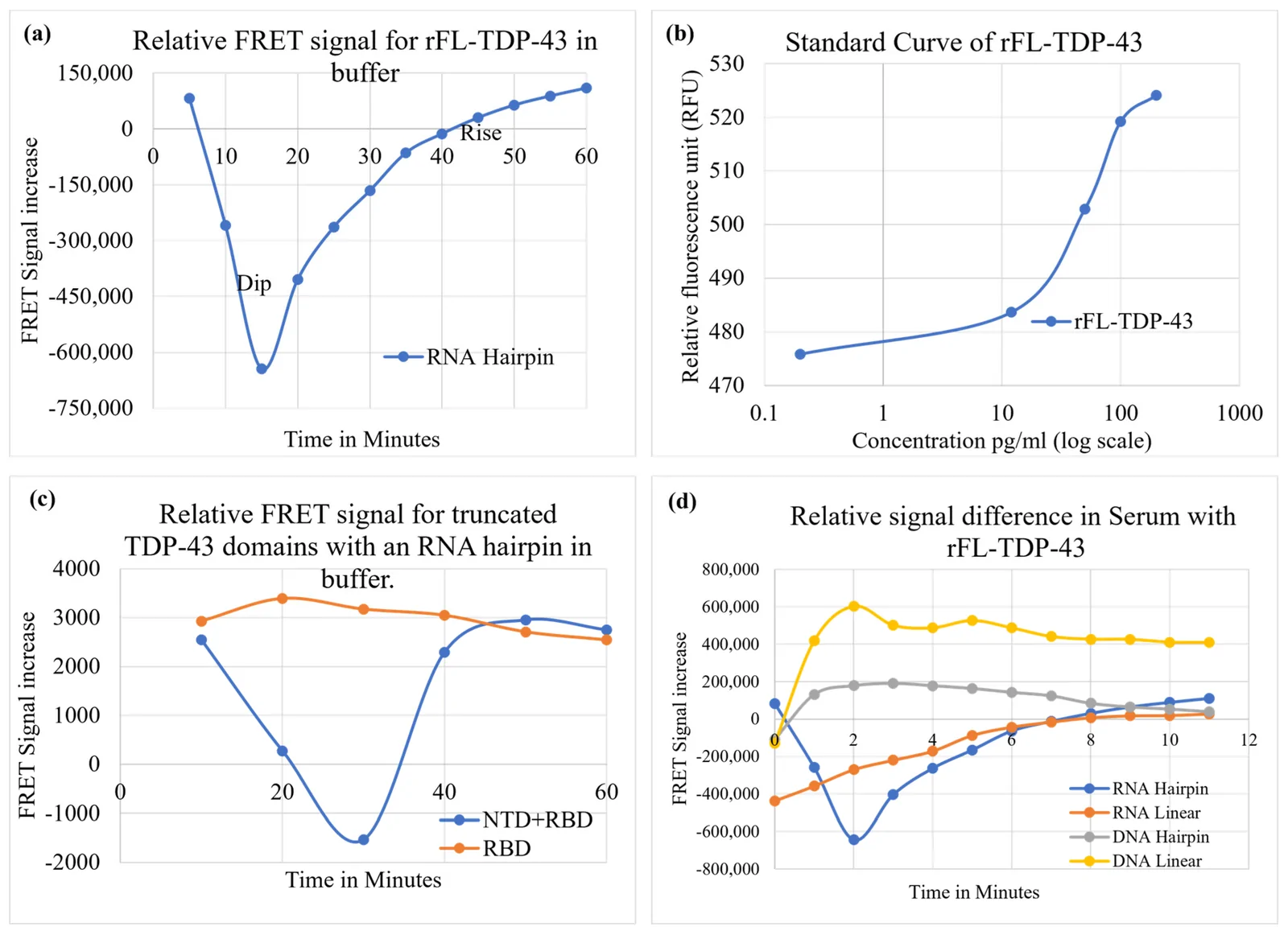
Kinetic dynamics, concentration scaling, domain mapping, and substrate specificity of TDP-43 hTR-FRET: (**a**) Biphasic “dip-then-rise” kinetic signature of rFL-TDP-43 with RNA hairpin in buffer. (**b**) Dynamic concentration–response curve of rFL-TDP-43 (0.2–200 pg/mL). (**c**) Domain mapping of truncated constructs (NTD + RBD vs. RBD) revealing NTD dependency for the initial dip phase. (**d**) Substrate and topological specificity comparing RNA versus DNA hairpins and linear probes in serum. Data represent mean ± SEM (n=3).

This precursor substrate comprised a “faux Exon 1” and “faux Exon 2” separated by a central “faux intron” loop, flanked by a 3’-biotin/terbium cryptate donor and a 5’-Atto 620 acceptor fluorophore. In its native folded state, spatial proximity between donor and acceptor fluorophores yielded a high baseline TR-FRET signal. Substrate cleavage or intramolecular reorganization physically separated the fluorophores, attenuating resonance energy transfer into solution. However, kinetic evaluation of this hairpin construct revealed a complex, biphasic “dip-then-rise” signature (Figure 2a), where an initial transient decrease was followed by a sustained, TDP-43-dependent signal increase. To eliminate potential biofluid nuclease susceptibility associated with the intron loop and isolate pure intermolecular bridging, we refined the assay configuration into a robust, linear dual-probe system (Figure 1b). The optimized platform employs two independent, Probe A and Probe B. In the absence of protein, free probes diffuse independently in solution, producing zero background FRET. To establish the atomic-level structural basis of probe binding, we performed tertiary structural modeling and 2D residue-level contact mapping using RNAproDB [19] (Figure 1c and Figure S3). Detailed residue interaction analysis of Probe A (“faux Exon 1”) confirmed dual-domain coordination across both RRM1 and RRM2 (Figure S3, left). When probe-competent TDP-43 engages Probe A and Probe B concurrently, it brings the fluorophores within the critical Förster distance ($R_{0}\approx 7\;\mathrm{nm}$), driving efficient non-radiative energy transfer and yielding a robust, long-lived emission at 665 nm (Figure 1d).

This final design employs two synthetic probes: a “faux exon 1” labeled with the acceptor fluorophore (λex 620 nm; λem 665 nm) and a “faux exon 2” labeled with the donor (terbium; λex 350 nm; λem 620 nm). Crucially, the faux intron sequence is omitted to enhance stability. In this linear setup, free substrates in solution cannot generate a FRET signal. A signal is generated exclusively when functional TDP-43 bridges these two probes, bringing the donor and acceptor within the Förster radius (r→R0 ≈ 7 nm). This proximity-dependent phase reflects donor–acceptor coupling mediated by probe alignment, serving as a functional fingerprint of active TDP-43–RNA engagement.

While recombinant TDP-43 concentration–response curves confirm linear signal scaling ($R^{2}>0.98$ across 0.2 pg/mL to 200 pg/mL), endogenous biofluid TDP-43 comprises a complex mixture of full-length, truncated, and oligomeric species. The assay is therefore designed and reported as a ratiometric functional platform (a.u.), rather than an absolute mass concentration assay (Figure 2b). For kinetic characterization and domain mapping, we analyzed the second phase of the assay response. A monotonic rise in 665 nm emission beginning 5–15 min after incubation and plateauing by 60 min was observed only in the presence of probe-competent TDP-43. Kinetic modeling confirmed that this represents a first-order proximity process, with the amplitude of ∆FRET correlating directly with TDP-43 functional levels in serum; no non-TDP-43 control proteins recapitulated this characteristic curve. To elucidate the molecular requirements driving this biochemical activity, domain mapping was performed (Figure 2c). Recombinant constructs containing the N-terminal domain (NTD) and the tandem RNA-binding domain (RBD) fully retained the canonical biphasic (dip-then-rise) kinetic signature. Intriguingly, the RBD domain supported only the signal rise phase and failed to reproduce the initial dip, pointing to a critical structural role for the NTD sequence in mediating early conformational rearrangements or liquid–liquid phase separation (LLPS) dynamics.

Substrate specificity validation further confirmed that signal generation is strictly RNA-dependent (Figure 2d). In the presence of full-length TDP-43, RNA substrates containing canonical UU motifs produced the characteristic dip-then-rise signature, whereas DNA probes failed to elicit the biphasic kinetic signature (Figure 2d). Furthermore, while RNA hairpins generated the distinct dip followed by a gradual increase consistent with secondary structure stabilization, DNA hairpins showed only linear binding. Taken together, these results confirm that the measured hTR-FRET activity is strictly RNA-specific, topology-dependent, and relies on probe-competent, full-length TDP-43.

### 3.2. TDP-43 hTR-FRET Functional Activity as a Diagnostic Biomarker for ALS

We applied the hTR-FRET assay to a large cross-sectional cohort of serum samples. TDP-43 functional activity was significantly elevated in ALS patients (*n* = 905; mean = 390 ± 99 a.u.) compared to healthy controls (*n* = 145; mean = 302 ± 36 a.u.; *p* < 0.0001, Welch’s *t*-test) (Table 2).

This difference was demonstrated with a large effect size (Cohen’s d = 0.93) and good classification accuracy (AUC = 0.79, 95% CI: 0.76–0.82; *p* < 0.001). TDP-43 functional activity was quantified using the hTR-FRET assay, which demonstrated intra-assay precision of 4.4% CV and inter-plate CV of 15% (see Section 2.6). The ROC curve is presented in Figure S2. Baseline hTR-FRET values differed significantly across diagnostic groups (*p* < 0.0001). Cross-sectionally, TDP-43 activity discriminated ALS from healthy controls with a large effect size. It is important to note that population-level statistical separation (AUC, effect size) differs from individual diagnostic performance. The observed population-level separation warrants further validation before clinical application.

### 3.3. Genotype-Specific TDP-43 Functional Activity: Genetically Confirmed vs. Sporadic ALS

Table 2 summarizes hTR-FRET values across diagnostic groups. Healthy controls (*n* = 145) exhibited a mean of 302 ± 36 a.u., while sALS cases showed significantly higher activity (392 ± 99 a.u.; *p* < 0.001). Genetically confirmed ALS subtypes demonstrated distinct profiles: C9orf72 ALS averaged 382 ± 87 a.u., while SOD1 ALS showed lower activity (323 ± 10.5 a.u.), both prior to tofersen treatment. These findings suggest genotype-specific variation in extracellular, probe competent TDP-43 species detectable by the assay. Comparator groups exhibited values that overlapped with or exceeded ALS: FTD (458 ± 159 a.u.), AD-fast (443 ± 74 a.u.), and AD-slow (478 ± 200 a.u.), though these cohorts were small (*n* = 10–20). While differences versus ALS were not statistically significant (*p* = 0.059–0.256), the trend indicates potential disease-specific patterns requiring validation in larger cohorts.

### 3.4. Diagnostic Thresholds and Distribution Analysis of TDP-43 hTR-FRET Values

**Figure 3:**
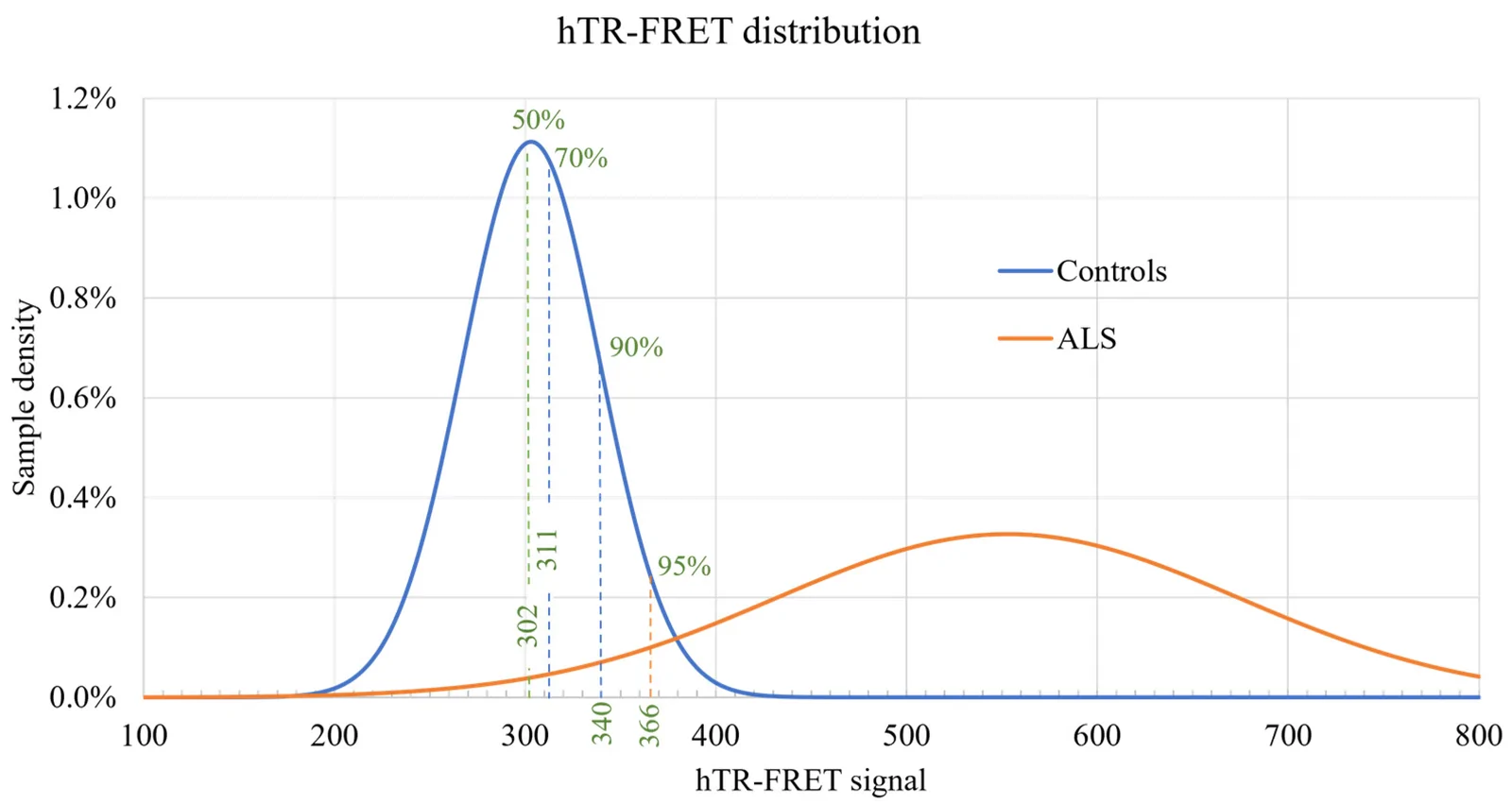
Distribution and diagnostic performance of TDP-43 hTR-FRET values in ALS and controls: ALS distribution shows the distribution of TDP-43 hTR-FRET value in ALS patients (*n* = 905) compared to healthy controls (*n* = 145). A 366 a.u. TDP-43 FRET value serves as the cutoff for 95% specificity. The large effect size (Cohen’s d = 0.93) indicates good separation between disease and healthy states, supporting the potential for population-level discrimination in research settings.

Figure 3 illustrates the distribution of hTR-FRET values in ALS patients (*n* = 905) versus healthy controls (*n* = 145). A cutoff of 366 a.u. achieves 95% specificity, effectively separating ALS from controls (sensitivity = 55.91%, PPV = 98.64%, NPV = 25.70%, AUC = 0.79 [95% CI: 0.76–0.82]; Supplementary Table S2). Kernel density curves highlight the distinct profiles: controls exhibit a narrow, approximately normal distribution centered at lower values, while ALS shows a broader, right-shifted distribution with higher means, reflecting elevated TDP-43 functional activity. Control-derived thresholds were established to define specificity against healthy controls for research purposes: 366 a.u. (95% specificity), 340 a.u. (90% specificity), 311 a.u. (70% specificity), and a median of 302 a.u. Diagnostic-group means are reported in Table 2. Clinical application requires prospective validation. The large effect size (Cohen’s d = 0.93) and group separation underscore the assay’s potential for population-level discrimination in research settings. Cutoff selection should be guided by the intended application (e.g., screening vs. confirmatory) in future prospective studies.

### 3.5. Diagnostic Group Comparison of TDP-43 hTR-FRET Activity

**Figure 4:**
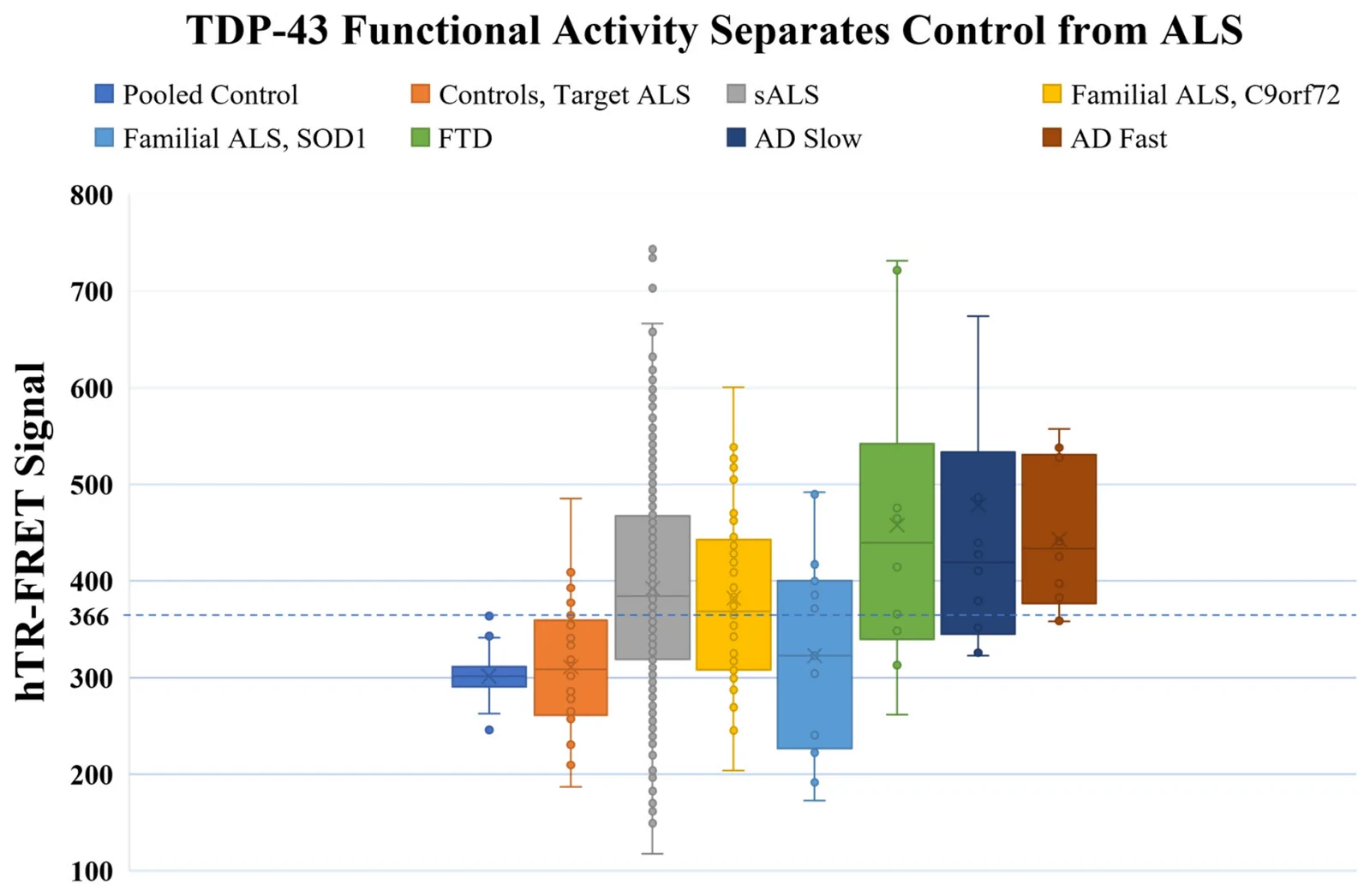
Cross-sectional hTR-FRET TDP-43 functional activity across diagnostic groups: Boxplots and overlaid kernel density estimates for pooled control, Target ALS controls, sALS, C9orf72 ALS, SOD1 ALS, FTD, AD-slow, and AD-fast. Each box shows median and interquartile range; whiskers extend to 1.5× IQR, and individual points represent samples that passed triplicate QC. Annotation: dashed vertical line at 366 a.u. marks the 95% control separation threshold.

Cross-sectional analysis of hTR-FRET TDP-43 signal revealed statistically significant group-level differences across diagnostic categories, with significant overlap between individual distributions (Figure 4). Healthy controls clustered tightly at lower values, establishing a baseline of minimal TDP-43 functional activity. In contrast, ALS samples were significantly right-shifted and more dispersed, indicating elevated and variable activity. The 95% control separation threshold (366 a.u.) effectively distinguished ALS from controls with high specificity against controls. Preliminary observations suggest C9orf72-ALS cases exhibit lower activity than sporadic ALS, consistent with potential genotype-specific differences in RNA dysregulation. AD and FTD samples showed higher values, supporting the assay’s ability to detect TDP-43-related dysfunction across neurodegenerative conditions.

### 3.6. Longitudinal Analysis Shows High Variability and General Stability

Longitudinal analyses of serial serum samples revealed variable slopes of TDP-43 hTR-FRET functional activity across cohorts. Across all cohorts, mean changes per visit were modest and did not reach statistical significance, reflecting substantial inter-individual variability. In smaller cohorts, such as NEALS Bio01 (9 subjects, 22 measurements; slope −1.16 ± 31.77 a.u./visit, *p* = 0.920), NEALS Bio03 (17 subjects, 48 measurements; slope −13.56 ± 124.99, *p* = 0.670), and NEALS AIM (25 subjects, 64 measurements; slope −6.35 ± 47.13, *p* = 0.516), slopes were near zero or negative. By contrast, larger cohorts demonstrated positive mean slopes suggestive of gradual increases in TDP-43 functional activity over time: NEALS Bio02 (36 subjects, 103 measurements; slope 9.59 ± 38.46, *p* = 0.149), Answer ALS (112 subjects, 341 measurements; slope 7.87 ± 54.13, *p* = 0.128), and Target ALS (18 subjects, 70 measurements; slope 14.99 ± 44.69, *p* = 0.185).

**Figure 5:**
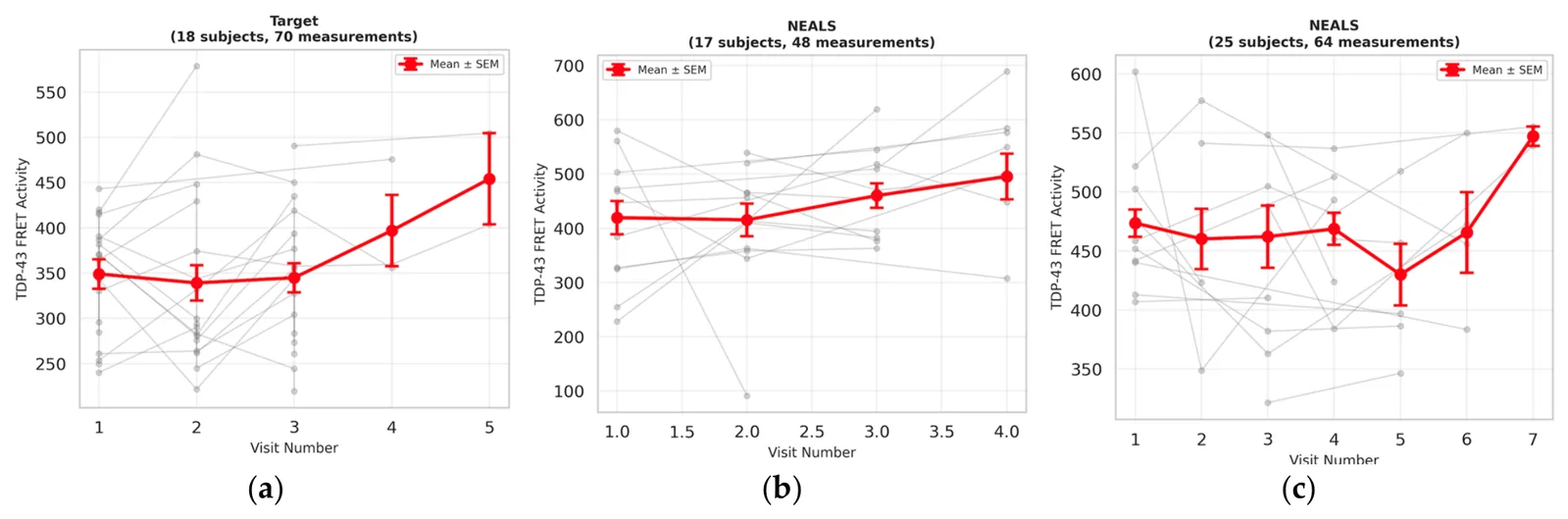
Longitudinal trajectories of TDP-43 functional activity: Temporal dynamics across serial sampling visits for three independent cohorts: (**a**) Target ALS cohort showing individual participant trajectories over visits; (**b**) NEALS Bio-02 cohort displaying temporal FRET changes; (**c**) NEALS Bio-03 cohort illustrating visit-to-visit variation.

These plots in Figure 5 depict the temporal dynamics of assay readings over multiple visits, revealing both cohort-level trends and substantial inter-individual variability. While mean trajectories suggest gradual increases in functional activity over time, the wide confidence intervals and divergent individual paths underscore the biological heterogeneity inherent in ALS progression.

**Figure 6:**
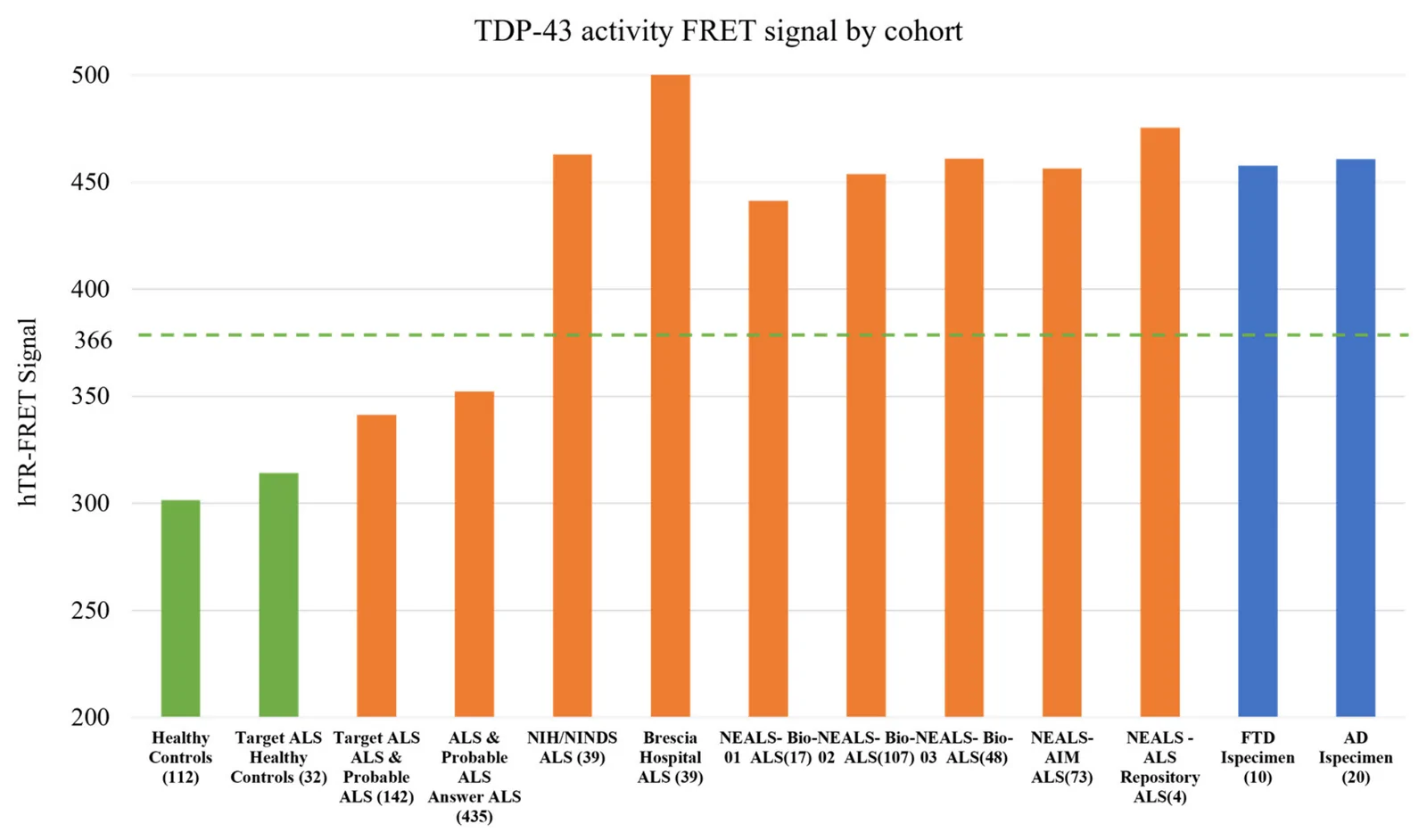
Cohort-level stratification of TDP-43 functional activity: Average TDP-43 functional activity FRET values for cohort and standard deviations. Bar graph showing hTR-FRET distributions stratified by Healthy Pooled Serum (HPS), healthy controls, ALS, FTD and AD in different colors and by contributing cohort of ALS samples (NEALS Bio01, NEALS Bio02, NEALS Bio03, NEALS AIM, NEALS ALS Repository, Answer ALS, Target ALS, NIH/NINDS, Spedali Civili Hospital of Brescia). Cohort-level bar chart indicates median and IQR; sample counts per subgroup annotated below each bar. Healthy Pooled Serum (HPS) was used strictly as an internal plate control for normalization, not as a biological cohort. AD-fast and AD-slow were defined based on clinical progression rates and cognitive decline trajectories reported in the source biorepository.

Although none of these trends achieved statistical significance. As illustrated in Figure 6, the largest cohorts (Target ALS, Answer ALS, NEALS Bio-02) show positive mean slopes consistent with modest increases in TDP-43 functional activity over time, but none reached statistical significance because of large inter-individual variability within each cohort. This consistent lack of a significant longitudinal trend suggests the TDP-43 signal is elevated at baseline and does not show a consistent trajectory across cohorts. For example, for 79 patients from the Answer ALS cohort whose time of death was reported, the time from disease onset to death ranged from 1.3 to 14.7 years, demonstrating substantial heterogeneity in survival duration for individuals living with ALS.

### 3.7. Lack of Consistent Correlation with Clinical Severity (ALSFRS-R)

**Figure 7:**
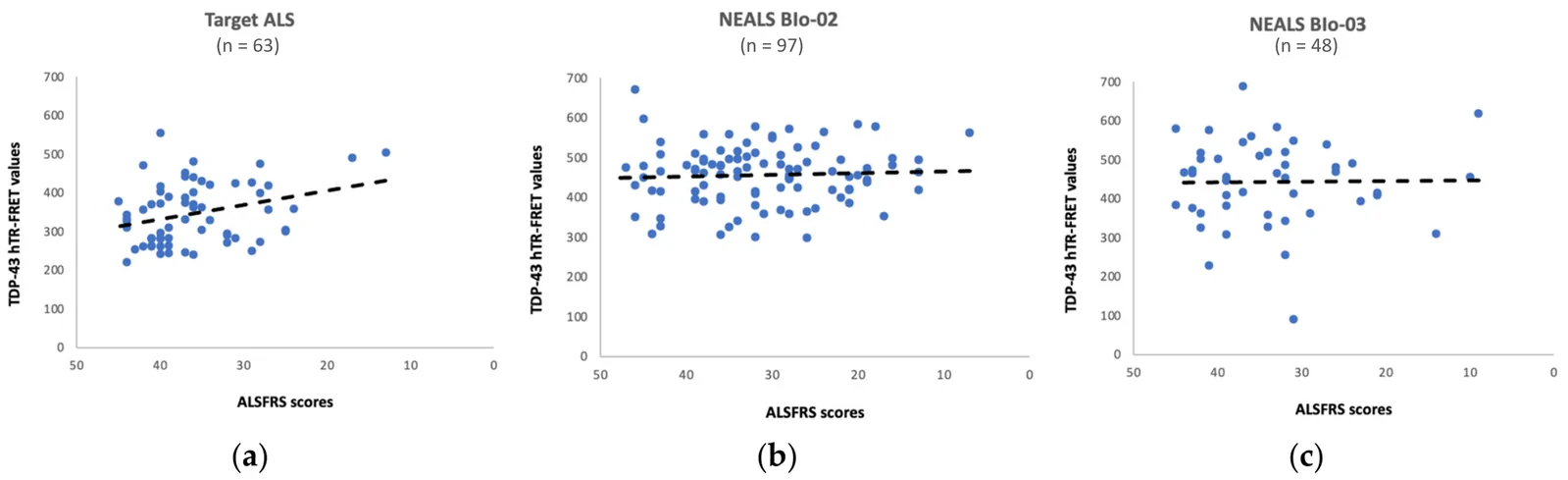
ALSFRS Correlations: Scatter plots showing correlation between baseline TDP-43 hTR-FRET signal (a.u.) and total ALSFRS-R scores: (**a**) Target ALS cohort, demonstrating a modest, statistically significant negative correlation; (**b**) NEALS Bio-02 cohort, showing a weak, non-significant negative trend; (**c**) NEALS Bio-03 cohort, showing no significant association.

We next correlated baseline TDP-43 functional activity with clinical severity using the ALSFRS-R score (Figure 7). Across the cohorts, there was no consistent, significant correlation between TDP-43 functional activity and ALSFRS score. For example, the NEALS—Bio-02 (*n* = 97) and NEALS—Bio-03 (*n* = 48) cohorts showed weak, non-significant negative trends (NEALS—Bio-02, r = −0.052, *p* = 0.613; NEALS—Bio-03, r = −0.013, *p* = 0.928). An exception was observed in the Target ALS cohort (*n* = 63), which demonstrated a statistically significant, modest negative correlation (Pearson r = −0.3146, *p* = 0.012; Spearman rho = −0.2765, *p* = 0.0283) between ALSFRS-R and TDP-43 functional activity.

### 3.8. Weak Correlation in Answer ALS Highlights Cohort Heterogeneity

**Figure 8:**
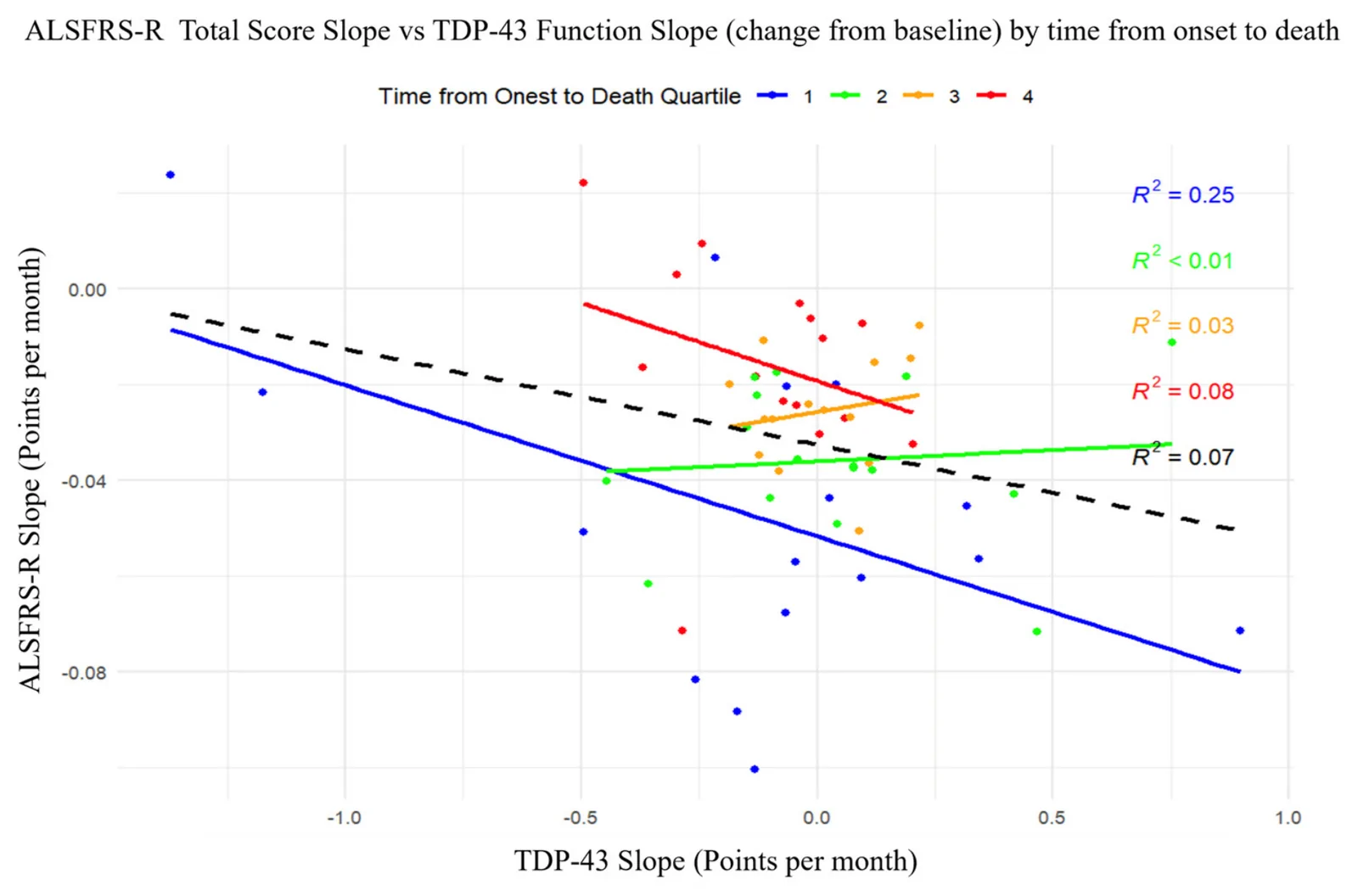
Correlation between functional decline and TDP-43 hTR-FRET values: Scatter plot of the ALSFRS-R decline rate (points/month) against the rate of change in TDP-43 functional activity (a.u./month), stratified by quartiles of time from symptom onset to death or last known follow-up. The analysis includes deceased participants with available paired longitudinal data. The overall inverse trend (black dashed line) reflects a modest association, with considerable inter-individual scatter, indicating that steeper increases in TDP-43 activity tend to coincide with faster functional decline. This association is most pronounced in the earliest disease quartile (Q1, blue; shortest time from onset), where the correlation is strongest (R^2^ = 0.25), suggesting a closer coupling between molecular dysregulation and clinical deterioration in rapid progressors.

Similarly, an exploratory correlation analysis was performed by correlating the rate of change in TDP-43 functional activity with the rate of functional decline (ALSFRS-R slope), and the results were highly variable. The largest cohort, Answer ALS (*n* = 134), showed a weak pseudo-longitudinal trend (overall R^2^ ≈ 0.07). This weak association was most pronounced in early-progression (defined as Quartile 1 based on time from symptom onset to death/last follow-up) individuals (Quartile 1, R^2^ = 0.25), (Figure 8).

The weak correlations likely reflect high biological heterogeneity and inclusion of atypical disease courses. This cross-sectional analysis does not constitute a prognostic study; findings are exploratory.

### 3.9. Paired Longitudinal Analysis of Paired TDP-43 Functional Activity and ALSFRS-R Scores in Answer ALS

**Figure 9:**
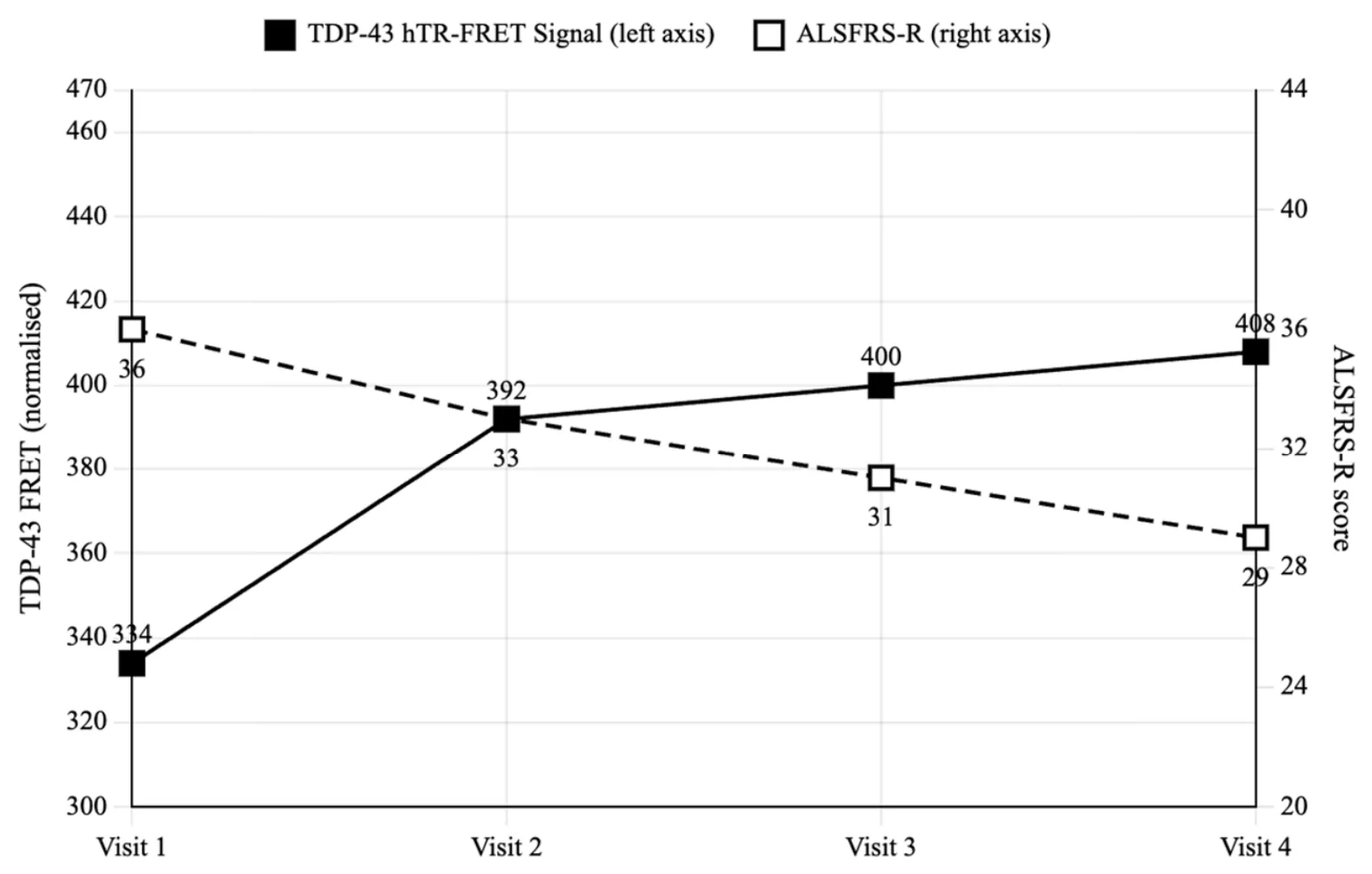
Longitudinal hTR-FRET values and ALSFRS-R scores stratified by disease progression quartiles: Longitudinal trajectories from Answer ALS participants illustrating changes in TDP-43 functional activity (a.u.) alongside ALSFRS-R scores over time. Data are stratified by quartiles of time from symptom onset to death or last follow-up to account for disease duration heterogeneity. Group-level trends show concurrent increases in TDP-43 activity and declines in motor function, though individual trajectories exhibit substantial variability. The figure highlights the exploratory nature of these associations and the high inter-individual scatter observed in longitudinal biomarker-clinical correlations.

A total of 134 Answer ALS participants were assessed, of whom 105 had concurrent baseline TDP-43 functional measurements and ALSFRS-R scores. A detailed demographic and longitudinal summary of this cohort is provided in Table S1. Demographics reflected typical ALS distributions (mean age of 60.5 years; 60.4% male; 88% sporadic) (Table S1). Participants were enrolled at a median of 1.7 years from symptom onset (range of 0.3–15.1 years) with baseline ALSFRS-R scores averaging 36 ± 7 and TDP-43 function at 358.9 ± 81 a.u. The baseline ALSFRS-R score of 36 ± 7 is consistent with mild to moderate clinical impairment. Between baseline and the next visit, TDP-43 functional activity showed a small mean increase from 358.9 ± 81.0 a.u. to 372.1 ± 92.3 a.u. (+8.9 ± 83.1 a.u.; *n* = 81), whereas ALSFRS-R scores declined by 6.8% ± 12.6% (*n* = 99) over the same interval. Group-level paired trends (↑ TDP-43, ↓ ALSFRS-R) suggest concurrent biochemical and clinical changes, though individual-level correlations remain modest and are influenced by inter-assay and biological variability. At the group level, rising TDP-43 activity was observed alongside declining ALSFRS-R scores, though individual trajectories showed high variability and modest correlation (Figure 9).

Genotype specific patterns were evident among 66 familial ALS samples. C9orf72 carriers exhibited a wide baseline range (≈204–600 a.u.), where higher early activity aligned with shorter survival (34–55 months), while lower or stable activity corresponded to longer survival (up to 65–68 months). SOD1 mutation carriers exhibited greater variability.

Participants with L114T, E100K, and c.374A>G mutations showed moderate TDP-43 functional activity with survival spanning 24–80 months. Notably, the sole SOD1 A4V carrier was a clear outlier, demonstrating low TDP-43 functional activity and preserved early ALSFRS-R despite rapid progression to death within 18 months.

## 4. Discussion

This study introduces a novel serum-based hTR-FRET assay that shifts the canonical TDP-43 biomarker development from static abundance to dynamic RNA-binding function. The functional readout discriminates ALS from controls with good classification accuracy (AUC = 0.79) and shows cohort-dependent exploratory trends, where higher activity associates with worse function and shorter survival. To our knowledge, this is the first demonstration that probe-competent TDP-43 functional state can be directly quantified in human serum. Specificity is achieved by engineering the synthetic RNA binders requiring TDP-43 engagement of (UU)n RNA motifs; signal arises only when motif-competent TDP-43 bridges labeled RNA probes, effectively functioning as a molecular logic gate. Using hTR-FRET with a long-lifetime lanthanide donor (τ^D^ ≈ 1.6 ms) suppresses serum autofluorescence, enabling picogram sensitivity. The biphasic “dip-then-rise” trace reflects a transient initial signal decrease followed by motif-dependent rise, reporting functional TDP-43 molecules. Serum was chosen over plasma, as plasma contains anticoagulants and fibrinogen, which reduce FRET efficiency and abolish the biphasic signature [16].

### 4.1. Diagnostic Performance and Genotype Stratification

In a large cross-sectional cohort, the assay detected significantly elevated functional activity in ALS serum versus controls (AUC = 0.79). Exploratory genotype stratification showed mean values of 392 a.u. for sporadic ALS, 382 a.u. for C9orf72 ALS, and 323 a.u. for SOD1 ALS. The subgroup patterns, particularly the distinct values in SOD1-ALS, suggest that TDP-43 functional activity in biofluids varies with genetic background and may reflect interactions between SOD1 pathology and TDP-43 dysfunction; however, studies need to be repeated in a larger cohort of familial ALS. Although SOD1 ALS has traditionally been considered outside the spectrum of TDP-43 proteinopathies, emerging but not yet consensus evidence suggests that misfolded SOD1 aggregates can induce TDP-43 mislocalization and aggregation. However, this remains an area of active investigation, and the causal directionality has not been established [20]. Additional studies have demonstrated co-deposition of SOD1 and phosphorylated TDP-43 in motor neurons of both familial and sporadic ALS cases, reinforcing the possibility of shared pathogenic cascades [21,22]. In our cohort, 15 genetically confirmed SOD1 ALS cases exhibited the hTR-FRET value (323 ± 10.5 a.u.), exceeding healthy controls. While exploratory due to limited sample size, these findings demonstrate that functional TDP-43 activity is detectable in SOD1 ALS serum and support the hypothesis that TDP-43 dysfunction may play a role even in genetically distinct ALS subtypes. The wide baseline range (≈204–600 a.u.) in C9orf72 carriers likely reflects disease heterogeneity, variable timepoints from onset, and potential differences in extracellular TDP-43 release kinetics. TDP-43 proteinopathy is a recognized hallmark in a substantial subset of FTD cases. The elevated signal likely reflects this co-pathology. In the context of emerging therapeutics targeting TDP-43 biology, a functional serum readout may prove useful for target engagement studies, pending prospective validation. The present findings are hypothesis-generating and do not yet constitute a validated pharmacodynamic tool.

### 4.2. Interpreting Elevated Serum TDP-43 Functional Activity

Elevated serum hTR-FRET values should not be conflated with restored nuclear splicing function. The assay detects extracellular, probe-competent TDP-43 species released during progressive neurodegeneration. As cellular integrity declines, TDP-43 is shed via exosomes, microvesicles, and cellular demise. A subset of these extracellular fragments preserves its tandem RNA-binding domain architecture, allowing them to bridge synthetic UU-rich probes. Thus, higher hTR-FRET amplitudes index the quantity of circulating, dysfunctional TDP-43 species rather than an intact nuclear splicing program. This distinction reconciles the apparent paradox between increased measurable activity in biofluids and the intracellular loss-of-function documented histopathologically [1,10,23]. While non-ALS comparator cohorts (AD, FTD) exhibited elevated signals overlapping with ALS, these findings align with the known presence of TDP-43 co-pathology in these neurodegenerative conditions. The assay captures broad TDP-43 proteinopathy rather than ALS-exclusive pathology, and future studies with larger, age-matched comparator cohorts are necessary to define differential diagnostic cutoffs.

Moreover, several biological mechanisms may explain this increase. Neurodegeneration releases intracellular contents, including full-length and truncated TDP-43 species, into biofluids; some of these extracellular species preserve RBD architecture and transiently bind UU-rich sequences [14,17]. Proteolytic cleavage and post-translational modifications produce a heterogeneous pool where a subset of molecules remains aggregation-prone but probe-competent. Furthermore, mislocalized cytoplasmic TDP-43 may participate in aberrant assemblies that paradoxically increase RNA-binding interface accessibility even as nuclear function collapses [24]. Therefore, a higher hTR-FRET amplitude likely indexes the quantity of probe competent TDP-43 species in serum rather than an intact splicing program. Rising extracellular TDP-43 may reflect progressive cellular release and accumulation of probe-competent species as neurodegeneration advances.

### 4.3. Longitudinal Trends and Potential as a Progression Biomarker

Longitudinal analysis revealed highly variable trajectories. Most longitudinal analyses were non-significant and exhibited substantial inter-individual variability. While the largest cohorts exhibited positive mean slopes, none reached statistical significance. These findings represent exploratory associations warranting prospective clinical study. C9orf72 carriers showed particularly pronounced early elevation that aligned with more aggressive disease and reduced survival, though larger cohorts are required to validate stratification utility. SOD1 cases largely followed similar trends (*n* = 14/15 samples), but mutation-specific outliers (for example, an A4V carrier with rapid decline despite low serum activity) highlight that some pathogenic trajectories are TDP-43-independent.

This provides the first direct biofluid evidence supporting TDP-43 as a potential progression biomarker and aligns with models implicating extracellular TDP-43 in disease propagation [21,22,25]. While exploratory genotype patterns are noted, larger cohorts are required to validate stratification utility. The present findings are hypothesis-generating and do not yet constitute a validated pharmacodynamic tool. Baseline TDP-43 functional activity correlated negatively with ALSFRS-R in the Target ALS cohort (r=−0.31, p=0.012), but this association was not replicated across all samples of the Answer ALS cohort, emphasizing the impact of pre-analytical variability. Factors such as sample age, freeze–thaw cycles, clotting time, and collection protocols can influence protein solubility and assay readouts [13]. We therefore interpret inter-cohort value differences cautiously and recommend harmonized pre-analytic SOPs and prospective stability studies to disentangle true biological signals from handling artifacts. While NEALS employs standardized SOPs, differences in site-specific handling, storage duration, and assay batch effects can still introduce variability. Harmonization remains a key recommendation for future multi-cohort integration. NEALS SOPs represent best practice but were not uniformly applied in all samples (e.g., iSpecimen-sourced AD/FTD comparator samples). A formal pre-analytic stability study is proposed as a priority for the next phase. A formal discovery/replication design is required to resolve cohort-specific slope differences, which may reflect assay noise and limited sample sizes rather than true biological differences. The weak correlations likely reflect high biological heterogeneity, atypical disease courses, and pre-analytic variability, which mask clearer associations. Longitudinal associations remain exploratory and highly variable, warranting prospective validation. SOD1 pathology may indirectly influence TDP-43 dynamics, but mutation-specific outliers (e.g., A4V) highlight TDP-43-independent progression pathways. At the group level, paired trends (↑ TDP-43, ↓ ALSFRS-R) suggest concurrent biochemical and clinical changes, though individual-level correlations remain modest. High biological heterogeneity, pre-analytic variability, and atypical disease courses likely mask clearer associations. Longitudinal associations remain exploratory and warrant prospective validation.

In summary, elevated hTR-FRET value in ALS most plausibly reflects an increased burden of dysfunctional extracellular, probe-competent TDP-43 species. In the context of emerging therapeutics targeting TDP-43 biology, a functional serum readout may prove useful for target engagement studies, pending prospective validation.

## 5. Conclusions

The hTR-FRET TDP-43 functional assay represents a novel bioanalytical approach that transitions from measuring the static presence of a pathological protein to quantifying its dynamic biochemical RNA-bridging function. By integrating sophisticated RNA-probe design with highly sensitive hTR-FRET detection, we have developed a serum-based assay that demonstrates moderate group-level discrimination of ALS from controls and exploratory associations with disease trajectory, providing a proof-of-concept platform warranting prospective clinical study.

## Figures and Tables

**Table 1:** Serum distribution by diagnostic category: Tabulated breakdown of diagnostic categories, subgroup genotypes, sample counts, sources, and key notes on cohort composition.

Diagnostic Category	Specific Group/Genotype	Sample Count (Total *n* = 1080)	Source(s)
Control	Healthy Controls	145	Research donors from established biobanks (NEALS, NIH/NINDS, iSpecimen)
Amyotrophic Lateral Sclerosis (ALS)	Sporadic ALS (sALS)	839	NEALS (AIM, Bio1, Bio2, Bio3, ALS biorepository), NIH/NINDS, Spedali Civili Hospital of Brescia, Answer ALS, TARGET ALS.
	Familial ALS (fALS): • C9ORF72-ALS (*n* = 51) • SOD1-ALS (*n* = 15)	66	
Other Neurodegenerative Diseases (Comparator Group)	Frontotemporal Dementia (FTD) and FTD-GRN	10	iSpecimen
	Alzheimer’s Disease (AD)	20	

Note: Detailed demographic characteristics (age, sex, ethnicity, race) and longitudinal metrics for the Answer ALS cohort are provided in Table S1.

**Table 2:** Summary of cross-sectional TDP-43 hTR-FRET values.

	Number	Mean TDP-43 *	vs. Healthy	vs. ALS
Healthy Control	145	302 ± 36		
ALS (sALS)	839	392 ± 99	*p* < 0.001	
C9orf72-ALS	51	382 ± 87	*p* < 0.001	
SOD1-ALS	15	323 ± 10.5	*p* < 0.001	
FTD	10	458 ± 159	*p* < 0.001	*p* = 0.086 (ns)
AD Fast	10	443 ± 74	*p* < 0.001	*p* = 0.059 (trend)
AD Slow	10	478 ± 200	*p* < 0.001	*p* = 0.256 (ns)

* Values reported as mean ± SD.

## Data Availability

Details of the probe sequences and assay design are disclosed in the patent applications WO2024115914A1 and EP4347873A1. Source data for the Supplementary Materials are included online. Supplementary Materials contains the data underlying Table S1, and Supplementary Materials contains the data underlying Figure S1.

## Supplementary Materials

The following are available online at https://www.mdpi.com/article/10.3390/bios16080446/s1, Figure S1: Excitation and emission spectral profiles and optical wavelength optimization for hTR-FRET detection; Figure S2: Diagnostic performance of hTR‑FRET TDP‑43 functional activity; Figure S3: Structural interaction mapping of Probe A and Probe B with TDP-43 RRM domains; Table S1: Longitudinal tdp-43 functional activity: summary on answer ALS cohort; Table S2: Diagnostic Performance Metrics across Cutoff Thresholds.
