# Supplementary material for: Functional Activity of TDP-43: A Direct Biomarker for ALS
Source: Biosensors (Basel). 2026 Aug 17;16(8):446. doi: 10.3390/bios16080446 (PMC13511632; doi:10.3390/bios16080446)
Supplement: Supplementary file 1 [file biosensors-16-00446-s001.zip › biosensors-4447511-supplementary.pdf]

Figure S1: Excitation and emission spectral profiles and optical wavelength optimization for hTR-FRET detection: Excitation and emission spectra recorded for negative control (unbound donor/background baseline, red) and positive control (target-bound assembly exhibiting energy transfer, green) conditions. While the Atto 620 acceptor dye exhibits peak fluorescence emission near 640 nm (99.8% relative emission efficiency), direct donor excitation overlap (acceptor absorption) at this wavelength is 31.0%, introducing significant direct-excitation background noise. To eliminate spectral bleed-through and direct acceptor excitation, emission detection was optimized to $\lambda = 665$ nm. At 665 nm, Atto 620 retains 63.2% emission efficiency while direct acceptor absorption drops to 0.8%, achieving a $\sim$ 24.5-fold enhancement in the signal-to-background ratio (665 nm vs. 620 nm donor ratiometric readout).
RFU: Relative Fluorescence Units; Ex: Excitation spectrum; Em: Emission spectrum.

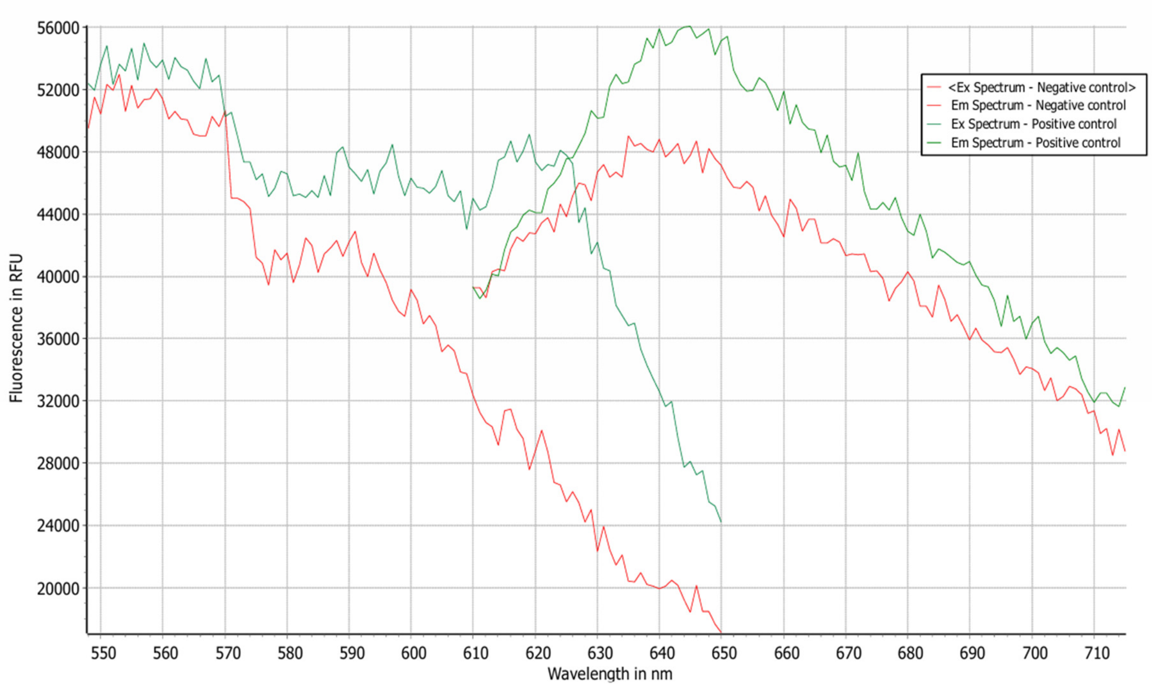

Figure S2: Diagnostic performance of hTR-FRET TDP-43 functional activity: Receiver operating characteristic curve for discrimination of ALS versus controls using hTR-FRET TDP-43 functional activity as the predictor (AUC = 0.79, 95% CI: 0.76–0.82; $p < 0.001$). Group means were computed from all cross-sectional samples; Cohen's d and p-value are reported on the figure (Samples are from mixed population, different prospectively cohort, and different time point).

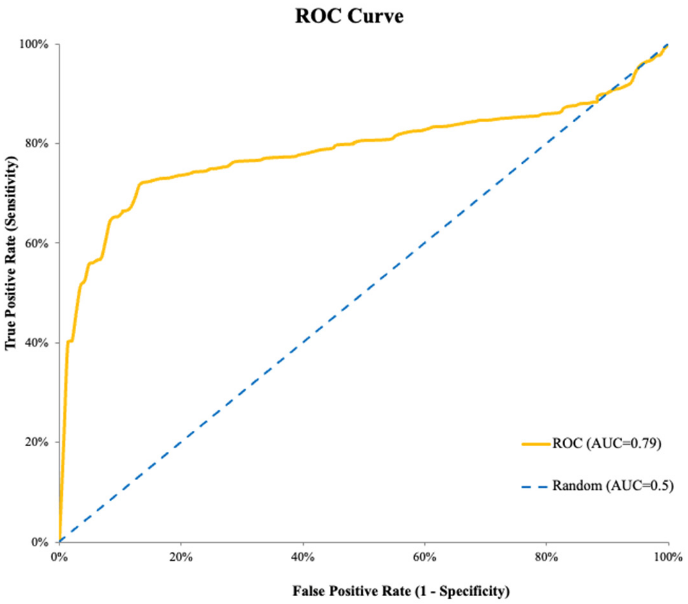

Figure S3. Structural interaction mapping of Probe A and Probe B with TDP-43 RRM domains: (Left) 3D cartoon structure and 2D interaction map of Probe A engaging TDP-43 tandem RNA recognition motifs (RRM1 and RRM2). (Right) 3D model and 2D residue-level contact map of the UU-rich Probe B bound across RRM1 and RRM2, highlighting key amino acid interactions. Generated using RNAproDB.

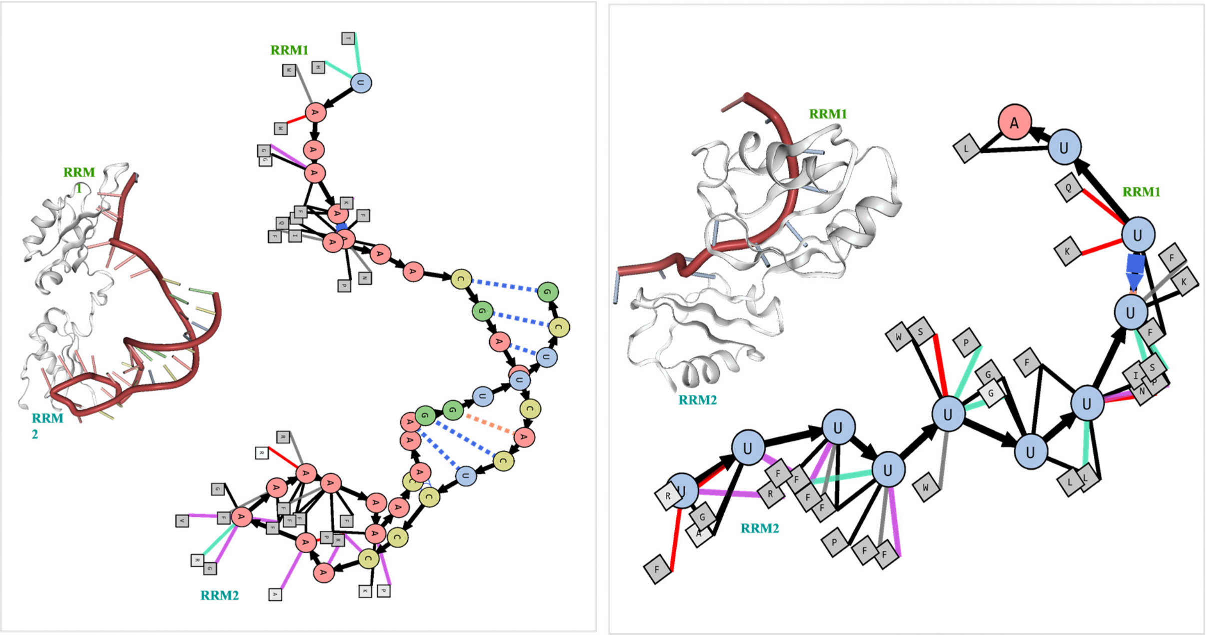

Table S1. Longitudinal tdp-43 functional activity: summary on answer ALS cohort			
Characteristic	All<br>(n = 134)	Any<br>(n = 131)	Baseline tdp-43<br>& baseline alsfrs-r*<br>(n = 105)
Age (years)			
Mean (sd)	60.5 (10.7)	60.6 (10.5)	61.0 (10.5)
Median	61.4	61.5	61.8

Range	32.0 - 85.3	32.0 - 85.3	33.1 - 85.3
Sex, n (%)			
Male	81 (60.4%)	78 (59.5%)	65 (61.9%)
Female	53 (39.6%)	53 (40.5%)	40 (38.1%)
Ethnicity, n (%)			
Hispanic/latino	7 (5.2%)	6 (4.6%)	5 (4.8%)
Not hispanic/latino	127 (94.8%)	125 (95.4%)	100 (95.2%)
Race, n (%)			
Black/african american	4 (3%)	4 (3.1%)	3 (2.9%)
White	127 (94.8%)	124 (94.7%)	100 (95.2%)
Other	2 (1.5%)	2 (1.5%)	1 (1%)
Als group, n (%)			
Fals	16 (11.9%)	16 (12.2%)	13 (12.4%)
Sals	118 (88.1%)	115 (87.8%)	92 (87.6%)
Time from onset to baseline (years)*			
N	132	130	104
Mean (sd)	2.4 (2.2)	2.4 (2.2)	2.5 (2.3)
Median	1.7	1.7	1.7
Range	0.3 - 15.1	0.3 - 15.1	0.3 - 15.1
Time from diagnosis to baseline (years)*			
N	133	131	105
Mean (sd)	1.0 (1.6)	1.0 (1.6)	1.1 (1.8)
Median	0.4	0.4	0.5
Range	0 - 13.7	0 - 13.7	0 - 13.7
Elescrlr, n (%)			
N	132	131	105
Definite	43 (32.6%)	43 (32.8%)	37 (35.2%)
Possible	20 (15.2%)	20 (15.3%)	17 (16.2%)
Probable	34 (25.8%)	33 (25.2%)	28 (26.7%)
Probable laboratory supported	31 (23.5%)	31 (23.7%)	21 (20%)
Suspected	4 (3%)	4 (3.1%)	2 (1.9%)
Onset by cns region, n (%)			
Bulbar			
Yes	34 (25.4%)	34 (26%)	27 (25.7%)
No	100 (74.6%)	97 (74%)	78 (74.3%)
Axial			
Yes	8 (6%)	8 (6.1%)	7 (6.7%)
No	126 (94%)	123 (93.9%)	98 (93.3%)
Limb			
Yes	99 (73.9%)	97 (74%)	78 (74.3%)
No	35 (26.1%)	34 (26%)	27 (25.7%)
Other			

Yes	2 (1.5%)	2 (1.5%)	1 (1%)
No	132 (98.5%)	129 (98.5%)	104 (99%)
Tdp-43 function value (baseline*)			
N	109	109	105
Mean (sd)	360.3 (83.0)	360.3 (83.0)	358.9 (81.0)
Median	335	335	334
Range	215.0 - 594.0	215.0 - 594.0	215.0 - 552.0
Tdp-43 function value (visit 2)			
N	101	101	81
Mean (sd)	367.1 (91.3)	367.1 (91.3)	372.1 (92.3)
Median	352	352	353
Range	166.0 - 748.0	166.0 - 748.0	178.0 - 748.0
Change in tdp-43 function value baseline to visit 2			
N	85	85	81
Mean (sd)	8.8 (83.6)	8.8 (83.6)	8.9 (83.1)
Median	2	2	2
Range	-466	-466	-449
Alsfrs-r total score (baseline*)			
N	129	126	105
Mean (sd)	35.9 (7.3)	36.0 (7.2)	35.6 (7.4)
Median	38	38	37
Range	12.0 - 47.0	12.0 - 47.0	12.0 - 47.0
King's stage (baseline*)			
Stage 0	5 (3.7%)	5 (3.8%)	0 (0%)
Stage 1	34 (25.4%)	34 (26%)	26 (24.8%)
Stage 2	30 (22.4%)	30 (22.9%)	24 (22.9%)
Stage 3	31 (23.1%)	29 (22.1%)	25 (23.8%)
Stage 4a	2 (1.5%)	2 (1.5%)	2 (1.9%)
Stage 4b	32 (23.9%)	31 (23.7%)	28 (26.7%)
% change in ALSFRS_R total score from baseline to visit 2*			
N	99	99	99
Mean (sd)	-6.8 (12.6)	-6.8 (12.6)	-6.8 (12.6)
Median	-5	-5	-5
Range	-73.1	-73.1	-73.1
Genetic mutations, n (%)			
Ang			
N	6	6	5
Negative	6 (100%)	6 (100%)	5 (100%)
Positive	0 (0%)	0 (0%)	0 (0%)
C9orf72			
N	42	42	34
Negative	32 (76.2%)	32 (76.2%)	26 (76.5%)
Positive	10 (23.8%)	10 (23.8%)	8 (23.5%)

Fus			
N	7	7	5
Negative	7 (100%)	7 (100%)	5 (100%)
Positive	0 (0%)	0 (0%)	0 (0%)
Progran			
N	3	3	3
Negative	3 (100%)	3 (100%)	3 (100%)
Positive	0 (0%)	0 (0%)	0 (0%)
SETX			
N	6	6	5
Negative	5 (83.3%)	5 (83.3%)	5 (100%)
Positive	1 (16.7%)	1 (16.7%)	Na
SOD1			
N	12	12	10
Negative	7 (58.3%)	7 (58.3%)	6 (60%)
Positive	5 (41.7%)	5 (41.7%)	4 (40%)
Positive	0 (0%)	0 (0%)	0 (0%)
**Baseline assumed to be visit 1 value reported as mean ± sd*<br>*Note: specific SOD1 mutations identified in this cohort include L114t, E100K, and C.374 A>G. SETX variants are pending formal pathogenicity confirmation per clinical genetics reporting standards. All familial ALS cases were confirmed genetically positive through clinical diagnostic panels prior to biorepository inclusion.*			

Table S2. Diagnostic Performance Metrics across Cutoff Thresholds.:
TDP-43 hTR-FRET values across predefined and Youden-optimized thresholds. Performance metrics along with 90% Confidence Intervals (Wilson score method) for Sensitivity, Specificity, Positive Predictive Value (PPV), and Negative Predictive Value (NPV) across candidate cutoffs. The overall diagnostic performance across the entire cohort yields an Area Under the Curve of AUC = 0.788 (90% CI: 0.76–0.81).

Cutoff Threshold (a.u.)	Specificity % (90% CI)	Sensitivity % (90% CI)	PPV % (90% CI)	NPV % (90% CI)
366.0 a.u. (95% Spec Cutoff)	95.2% (91.3%–97.4%)	56.0% (53.3%–58.7%)	98.6% (97.5%–99.3%)	25.7% (22.8%–29.0%)
340.3 a.u. (90% Spec Cutoff)	90.3% (85.5%–93.7%)	65.5% (62.9%–68.1%)	97.7% (96.5%–98.5%)	29.6% (26.1%–33.3%)
317.0 a.u. (75% Spec Cutoff)	75.2% (68.8%–80.6%)	75.0% (72.6%–77.3%)	95.0% (93.4%–96.1%)	32.5% (28.5%–36.9%)
310.8 a.u. (70% Spec Cutoff)	69.7% (63.1%–75.5%)	76.7% (74.3%–78.9%)	94.0% (92.4%–95.3%)	32.4% (28.2%–36.9%)

302.0 a.u. (50% Spec Cutoff)	50.3% (43.6%–57.1%)	80.3% (78.1%–82.4%)	91.0% (89.2%–92.5%)	29.1% (24.6%–34.0%)

Note: Overall, ROC AUC = **0.788** (90% CI: 0.76–0.81). PPV and NPV are calculated based on cohort sample proportions ($N_{\text{ALS}} = 905$, $N_{\text{Controls}} = 145$) and are intended for research cohort comparison.
